# ViTKAB: an efficient deep learning network for cotton leaf disease identification

**DOI:** 10.3389/fpls.2025.1719877

**Published:** 2025-12-11

**Authors:** Laixiang Xu, Hongyun Song, Xiaodong Yang, Peng Xu, Zhaopeng Cai

**Affiliations:** 1 School of Computer and Data Science, Henan University of Urban Construction, Pingdingshan, China; 2 Institute of Food Science and Technology, Chinese Academy of Agricultural Sciences, Beijing, China; 3 College of Engineering, Key Laboratory of Modern Agricultural Equipment of Jiangxi Province, Jiangxi Agricultural University, Nanchang, China; 4 Binary Graduate School, Binary University of Management & Entrepreneurship, Puchong, Selangor, Malaysia

**Keywords:** crop diseases, cotton leaf, deep learning, vision transformer, BiFormer

## Abstract

**Introduction:**

Cotton is a vital global economic crop and textile material, yet its yield and quality are threatened by leaf diseases such as brown spot, verticillium wilt, wheel spot, and fusarium wilt.

**Methods:**

We propose ViTKAB, a cotton disease recognition model based on an enhanced Vision Transformer that integrates a Kolmogorov-Arnold network and a BiFormer module. The model optimizes the Vision Transformer architecture to improve inference speed, employs nonlinear feature representation to better capture complex disease characteristics, and incorporates sparse dynamic attention to enhance robustness and accuracy.

**Results:**

Experiments show that ViTKAB achieves an average recognition accuracy of 98.05% across four cotton leaf diseases, outperforming models such as CoAtNet-7, CLIP, and PaLI.

**Conclusions:**

This method offers valuable insights for advancing intelligent crop disease detection systems and exhibits strong potential for deployment on edge devices.

## 1 Introduction

Cotton, a globally significant economic crop, faces substantial threats from diseases and pests throughout its growth cycle (Goyal et al., 2025). Accurate pest identification remains challenging for farmers due to specialized knowledge requirements and pest diversity, often leading to delayed interventions and yield losses. Current diagnostic methods field observations and remote consultations suffer from subjectivity, while laboratory techniques, though accurate, are time-consuming and impractical for rapid response. Early and precise detection is therefore essential for sustainable cotton production.

However, leaf imaging is affected by uneven illumination and complex backgrounds (George et al., 2025). Lighting variations cause shadows that obscure symptomatic areas, and weeds with similar pixel characteristics reduce target-background contrast. These factors challenge conventional image processing methods, often leading to suboptimal segmentation and limited recognition accuracy.

Traditional identification relying on expert observation is labor-intensive, subjective, and inefficient especially in remote areas lacking timely expertise. Machine learning and deep learning offer promising alternatives (Aldakheel et al., 2024). While convolutional neural networks (CNNs) show potential, they require large annotated datasets, which are costly and scarce due to symptom variability across growth stages. This data limitation constrains model robustness and generalization.

To address scarce labeled data, this study explores deep learning solutions incorporating data augmentation, transfer learning, and optimized architectures. These approaches enable robust feature learning from limited samples, supporting effective disease recognition without extensive manual annotation (Bala and Bansal, 2024). Based on the above analysis, we propose a deep learning-based method for cotton disease classification. The main contributions of this paper are as follows:

1. We designed an efficient embedding and feature aggregation approach for Vision Transformer, reducing its parameter count and computational complexity to enable faster inference on embedded and edge computing platforms.
2. We incorporated the BiFormer module to implement a sparse dynamic attention mechanism, which minimizes processing overhead by focusing computational resources on essential features while ignoring irrelevant background information.
3. We pioneered the use of the Kolmogorov-Arnold network for cotton disease recognition. By replacing standard linear transformations, KAN enhances the model’s ability to capture complex, multi-scale disease patterns, leading to significantly improved generalization.

The paper is organized as follows: Section 2 surveys literature on crop disease recognition, Section 3 describes the proposed methodology, Section 4 reports and analyzes experimental results, Section 5 discusses the implications, and Section 6 provides the conclusion.

## 2 Literature reviews

In recent years, some scholars have studied the extraction methods of plant disease leaves in the agricultural field. They used K-means clustering (Li et al., 2025), Otsu dynamic threshold segmentation (Zhou et al., 2021), and mathematical morphology (Song et al., 2025) to segment the disease image. These methods can only achieve effective segmentation of a single leaf spot against a complex background. It is still unable to segment accurately for the small lesions scattered across multiple leaves. Although watershed (Wang et al., 2022) can better separate overlapping speckle images, small structures and noises in the original image will form a pseudo minimum during processing, resulting in over-segmentation.

With the application of machine learning in agriculture, most methods use principal component analysis (PCA) and support vector machines (SVM) to process and classify plant disease leaves. For instance, Shi et al. (2025) presented an image processing technique based on a gray-level occurrence matrix and SVM for the automatic detection and treatment of leaf diseases in tomato crops. Its average accuracy was 89.5% for four diseases. Wei et al. (2025) described a recent molecular technique based on loop-mediated isothermal amplification for wheat pathogen detection. Xu and Wang (2025) suggested a new method based on the elliptical-maximum margin criterion of metric learning for the identification of wheat leaf diseases and their severity. Its identification accuracy was 94.8%. Ali et al. (2022) proposed a novel approach called feature fusion and PCA for crop disease identification and obtained an accuracy of 98.2%. Compared to ResNet50, its average accuracy increased by 1.69%. Rahman et al. (2022) devised an image processing method based on a gray-level co-occurrence matrix and SVM for the automatic detection and treatment of tomato leaf diseases. Its average accuracy was 92.5% for four kinds of tomato leaf diseases. Kumar et al. (2022) developed an adaptive SVM classifier for the detection of rice plant diseases like bacterial leaf blight, brown spots, and leaf smut. Its accuracy was up to 98.8% in detecting and classifying rice leaf diseases. Machine learning can meet the requirements of disease identification by using manual handling features to design a classifier (Han and Guo, 2024). However, the complex feature extraction project greatly affects work efficiency. It takes a lot of time to preprocess images and evaluate the effectiveness of features. The manual features are subjective, limited, and rough. Different plant types, growth stages, disease categories, and environmental lighting lead to complex disease symptoms and difficult feature extraction.

In the traditional methods of plant leaf disease identification, it is necessary to select and extract the characteristics of the disease artificially, which results in a lack of self-learning of disease characteristics. These methods are better for certain diseases, but their applicability is often suppressed, and the recognition effect is general when disease images are obtained under different backgrounds, weather conditions, and hazard levels. However, the deep learning method has been proven to have better performance in the identification of plant leaf diseases (Du et al., 2025). For instance, Li and Zhao (2025) built a novel approach, namely the GoogleNet and Resnet50 models, for the classification of cotton leaf diseases. It attained an average accuracy of 93.6%. It improved by 8.6% when compared to SVM. Elaraby et al. (2022) described a deep learning approach based on AlexNet for identifying 25 different types of wheat, cotton, grape, corn, and cucumber leaf diseases and produced an accuracy of 98.83%. Sin and Öztürk (2025) proposed an improved YOLOv5 network to detect unopened cotton bolls in the field accurately and with lower cost, which combined DenseNet, an attention mechanism, and Bi-FPN. Its average precision was 5.83% higher than that of YOLOv3 on their testing dataset. Priya et al. (2022) created an algorithm based on Faster R-CNN and GAN for 4000 cotton leaf disease detection. Soni and Gangwar (2025) evaluated the potential of three-band multispectral imagery for the detection of cotton leaf diseases and achieved an overall accuracy of 89%. Astani et al. (2022) classified 13 classes of tomato disease in farm and laboratory conditions with high accuracy and speed through ensemble classification and achieved an average accuracy of 95.98% on the PlantVillage. Moussafir et al. (2022) developed a hybrid model for tomato disease detection based on image data collection. It obtained an identification accuracy of 98.1% on the Plant Village dataset. Although deep learning algorithms achieved good results in crop disease detection (Gupta and Jadon, 2025), most of these studies combined transfer learning strategies with attention mechanisms. Deep learning models extract information from the entire image during the tomato leaf disease identification process (Girmaw and Muluneh, 2024). However, the useful features that need to be extracted only exist in the local area where the target object is located. In addition, the process of deep learning is not transparent. Even if the models produce a high accuracy score on the test set, they do not learn distinguishing features.

## 3 Proposed methods

### 3.1 Optimize vision transformer

**Figure 1:**
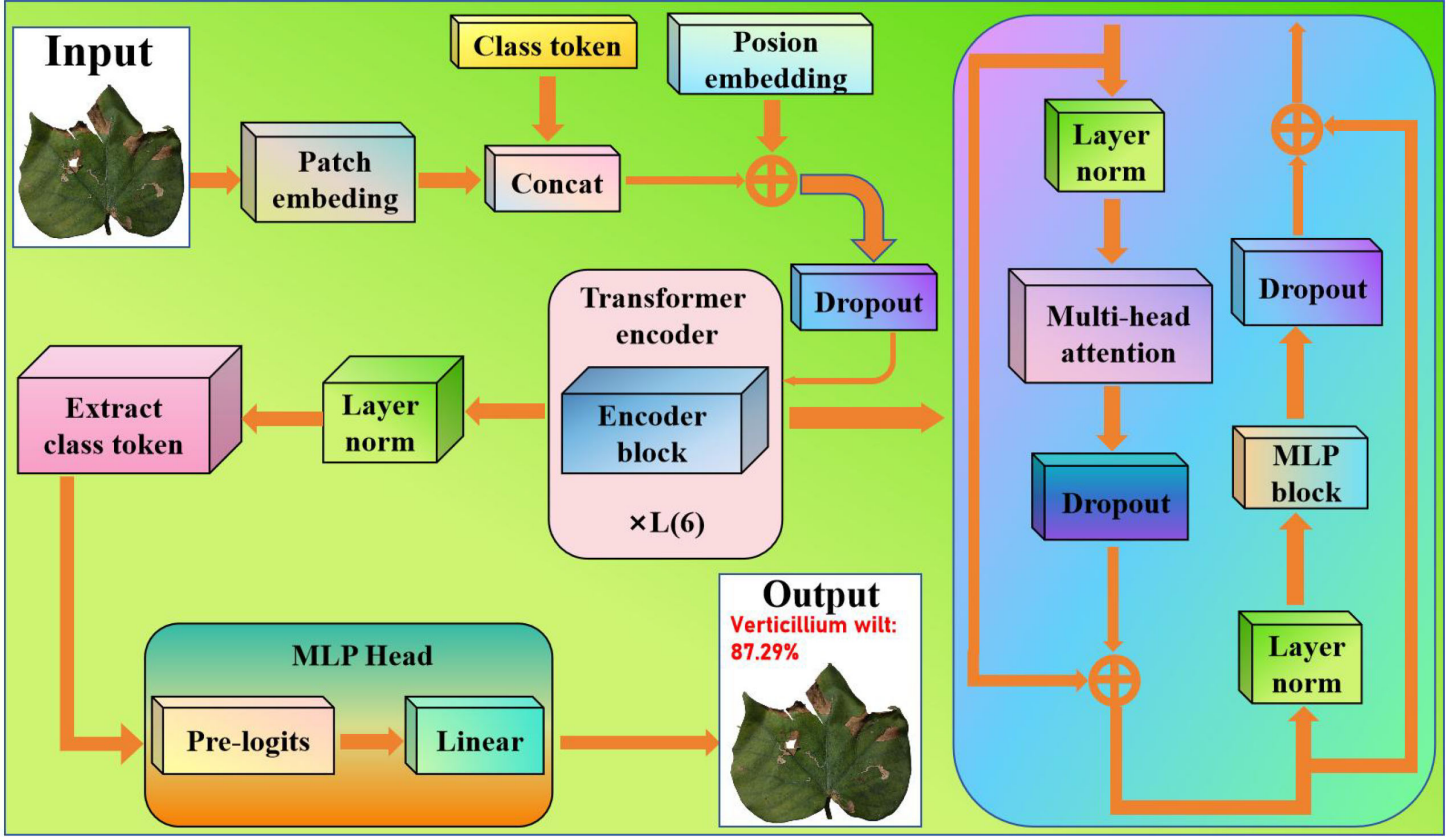
Improved vision transformer.

Given the sample size and complexity of cotton disease images, we used the standard Vision Transformer (ViT) as the basis network for feature extraction and classification. Cotton disease images include high dimensionality, diversity, and complicated local structures. ViT’s self-attention mechanism accurately models global dependencies and captures local-to-global characteristics, making it perfect for processing multi-scale and linked morphological information in cotton disease images. However, the original ViT model includes a large number of layers, which can easily lead to overfitting when training on tiny sample sets and increases computing cost, making it unsuitable for real-time recognition applications. **Figure 1** depicts the updated model, which includes a Patch Embedding layer, six Transformer layers, a Position Embedding layer, and a classification header (CLS Token).

As a result, we reduced the number of Transformer layers to six using the standard ViT. By reducing the number of model layers, the computational overhead was significantly lowered, which in turn greatly increased the image processing speed under identical hardware conditions. Despite this architectural simplification, the optimized ViT retains its core strength in modeling global feature dependencies. This enhancement grants the model greater adaptability for common devices, positioning it to meet the critical demand for real-time diagnostic efficiency in field applications. This enhancement meets the demand for feature extraction in cotton disease images by reducing model complexity and training difficulty while retaining global feature modeling capabilities, allowing lower-level features to better reflect cotton’s local morphology and texture patterns.

In the Transformer encoder, the previous layer’s feature sequence is transformed using the self-attention mechanism and MLP block, and the resulting result is normalized and residually connected to generate a new feature representation. The following describes how each layer’s output features are mapped to the input features. The input image is divided into N patches, linearly projected onto a d-dimensional vector, and then positional encoding is applied. It can be defined Equation 1:

(1) $$z_{0}=[X_{class};X_{p}^{1}E;X_{p}^{2}E;\ldots ;X_{p}^{N}E]+E_{pos}$$

where $\text{E}\in {\mathbb{R}}^{({\text{p}}^{2}\cdot \text{C})\times \text{d}}$ is the projection matrix, and $E_{pos}\in {\mathbb{R}}^{(N+1)\times d}$ is the position encoding.

Input feature sequence $X^{l-1}\in {\mathbb{R}}^{N\times d}$ (N is the number of patches + 1, and d is the embedding dimension) and generate Query, Key, and Value matrices through linear projection. They can be expressed Equations 2-4:

(2) $$\text{Q}={\text{X}}^{\text{l}-1}{\text{W}}_{\text{Q}},{\text{ W}}_{\text{Q}}\in {\mathbb{R}}^{\text{d}\times {\text{d}}_{\text{k}}}$$

(3) $$\text{K}={\text{X}}^{\text{l}-1}{\text{W}}_{\text{K}},{\text{ W}}_{\text{K}}\in {\mathbb{R}}^{\text{d}\times {\text{d}}_{\text{k}}}$$

(4) $$\text{V}={\text{X}}^{\text{l}-1}{\text{W}}_{\text{V}},{\text{ W}}_{\text{V}}\in {\mathbb{R}}^{\text{d}\times {\text{d}}_{\text{k}}}$$

where *l* is the layer number, $W_{Q}/W_{K}/W_{V}$ is the learnable weight matrix, and h is the number of attention heads.

The attention output can be calculated using Equation 5:

(5) $$Attention\;(Q,K,V)=soft\max(\frac{QK^{T}}{\sqrt{d_{k}}})V$$

The self-attention output is normalized by Layer Norm and input into an MLP block for nonlinear transformation. It can be formulated as Equation 6:

(6) $$X_{MLP}^{l}=GELU(X_{attn}^{l}W_{1}+b_{1})+W_{2}+b_{2}$$

where $W_{1}\in {\mathbb{R}}^{d\times 4d}$ and $W_{2}\in {\mathbb{R}}^{4d\times d}$ are the weight of a fully connected layer with an expansion ratio of 4.

Each layer’s output preserves the original information through residual connections. They can be expressed in Equations 7, 8:

(7) $$X^{l}=LayerNorm(X^{l-1}+DropPath(MSA(X^{l-1})))$$

(8) $$X^{l+1}=LayerNorm(X^{l}+DropPath(MLP(X^{l})))$$

where DropPath is a random depth regularization that discards the output of the sublayer with probability p during training.

Take the feature vector corresponding to the [CLS] tag and output the category probability through a single-layer linear layer. The process can be defined by Equation 9:

(9) $$y=soft\max(LayerNorm(X_{0}^{L})W_{head})$$

$X_{0}^{L}$ is the last layer [CLS] marker, $W_{head}\in {\mathbb{R}}^{d\times num\_classes}$.

Finally, the Transformer encoder extracts advanced semantic properties from images using many levels of feature transformation and information exchange, resulting in robust representation capabilities for downstream applications. This strategy not only preserves the crucial information in the input image, but it also increases the model’s ability to grasp global characteristics through the self-attention process.

### 3.2 Fused KAN

**Figure 2:**
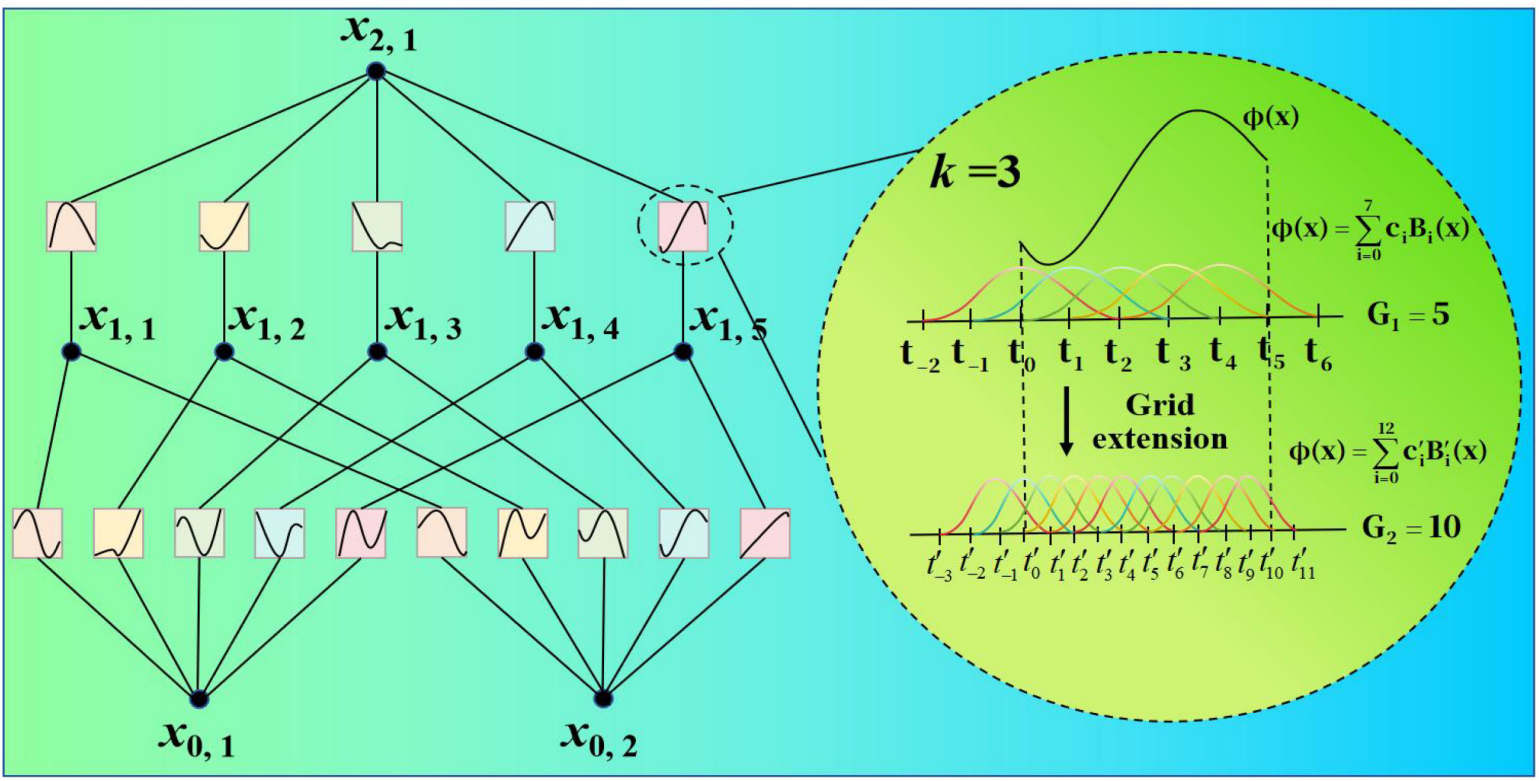
Proposed KAN.

Although the enhanced ViT decreases model complexity by reducing the number of Transformer layers while retaining global feature modeling capacity on small sample cotton disease images, there is still an issue with insufficient sensitivity to local fine-grained features. Cotton disease images show significant diversity and locally complex textures, and tiny morphological variations are important for classification outcomes. However, typical ViT has a limited ability to capture these local characteristics at shallow hierarchical structures, which can easily cause redundant information in the feature representation and interfere with cotton species classification. The activation function ReLU achieves straightforward yet effective nonlinearity by retaining positive values and suppressing negative ones. In contrast, the activation function GELU employs a probabilistic gating mechanism to smooth input features, thereby better capturing complex input patterns. Unlike traditional activation functions like ReLU and GELU, which have fixed forms, the proposed KAN learns activation functions and dynamically adapts to data, representing its core advantage. In agricultural image tasks, this flexibility allows the proposed KAN to more accurately model the intricate textures and irregular shapes of diseased leaves, significantly enhancing the model’s ability to identify subtle yet critical features. Such improved nonlinear expressiveness helps reduce feature redundancy, directs attention to discriminative local information for classification, and ultimately leads to higher classification accuracy. As a result, we introduce the KAN module with the goal of improving ViT. The proposed KAN structure is sketched in **Figure 2**.

Compared to the traditional Vision Transformer, the optimized KAN offers superior capabilities in nonlinear feature extraction. The standard ViT employs fixed, relatively simple nonlinear transformations, which can struggle to accurately model complex disease patterns. In contrast, the optimized KAN leverages learnable spline-based activation functions to dynamically shape complex, data-driven decision boundaries. This enables it to more precisely represent subtle features in disease imagery, such as gradual color shifts, fine textural variations, and irregular contours. This adaptive capability allows the model to isolate more discriminative features from complex backgrounds, significantly enriching feature representation and boosting classification accuracy. The KAN module provides exact optimization for the cotton feature extraction stage. After 6 layers of Transformer processing, the improved ViT has formed a preliminary global feature representation but still retains sufficient spatial information to reflect the local structure and morphological details of cotton. At this point, adding the KAN module can enhance attention allocation to key local patterns and multi-scale details, effectively suppress the interference of redundant features on classification, and enable the model to focus more on the core feature regions that distinguish cotton species, thereby improving classification performance and recognition accuracy.

For a smooth function ${\left[0,1\right]}^{n}\to \mathbb{R}$, it can be expressed by Equation 10:

(10) $$f(x)=\sum_{q=1}^{2n+1}\Phi_{q}(\sum_{p=1}^{n}\varphi_{q},p(x_{p}))$$

We parameterize the input features using B-spline basis functions. It can be expressed in Equation 11:

(11) $$f_{l,i,j(x_{j})}=\sum_{k=1}^{K}c_{l,i,j,k}B_{k}(x_{j};t)$$

where ${\text{B}}_{\text{k}}(\cdot ;\text{t}$) is the kth B-spline basis function (node vector t), ${\text{c}}_{\text{l},\text{i},\text{j},\text{k}}\in \mathbb{R}$ is the learnable coefficient, and K is the number of functions (default K = 5).

The input vector $\text{x}\in {\mathbb{R}}^{\text{n}}$ is projected through a set of univariate functions. It can be defined by Equation 12:

(12) $$z_{1,i}=\sum_{j=1}^{n}f_{1,i,j(x_{j})}$$

The output is ${\text{z}}_{1}\in {\mathbb{R}}^{\text{m}}$, where m is the width of the hidden layer.

The transformation of the *l*-th layer (l ≥ 2) can be calculated using Equation 13:

(13) $$\varphi_{l(z_{l-1})}=\sum_{i=1}^{m}g_{l,i(z_{l-1},i)}$$

The $g_{l,i(\cdot )}$ also adopts B-spline parameterization, reducing the number of parameters by sharing basis functions. The final output is a scalar that can be formulated Equation 14:

(14) $$y=\sum_{i=1}^{m}g_{\;L,i(z_{L-1,i})}$$

To prevent overfitting of univariate functions, we add a second-order derivative penalty term. It can be described by Equation 15:

(15) $$L_{\text{reg}}=\text{λ}\sum_{\text{l},\text{i},\text{j}}\int {\left(\frac{{\text{d}}^{2}{\text{f}}_{\text{l},\text{i},\text{j}}}{{\text{dx}}^{2}}\right)}^{2}\text{dx}$$

where λ is the trade-off coefficient (default=0.1).

Adaptively adjust network width based on input data. It can be expressed by Equation 16:

(16) $$m_{new}=\lfloor m_{init}.\frac{∥\nabla_{x}\varphi_{l}{∥}_{2}}{\alpha }\rfloor$$

where α is the gradient normalization constant.

By using a learnable univariate function in place of the fixed activation function used in classic MLP, along with B-spline parameterization and dynamic structural adjustment, KAN greatly increases the expressive capacity and parameter efficiency of the model.

### 3.3 Integrate BiFormer

**Figure 3:**
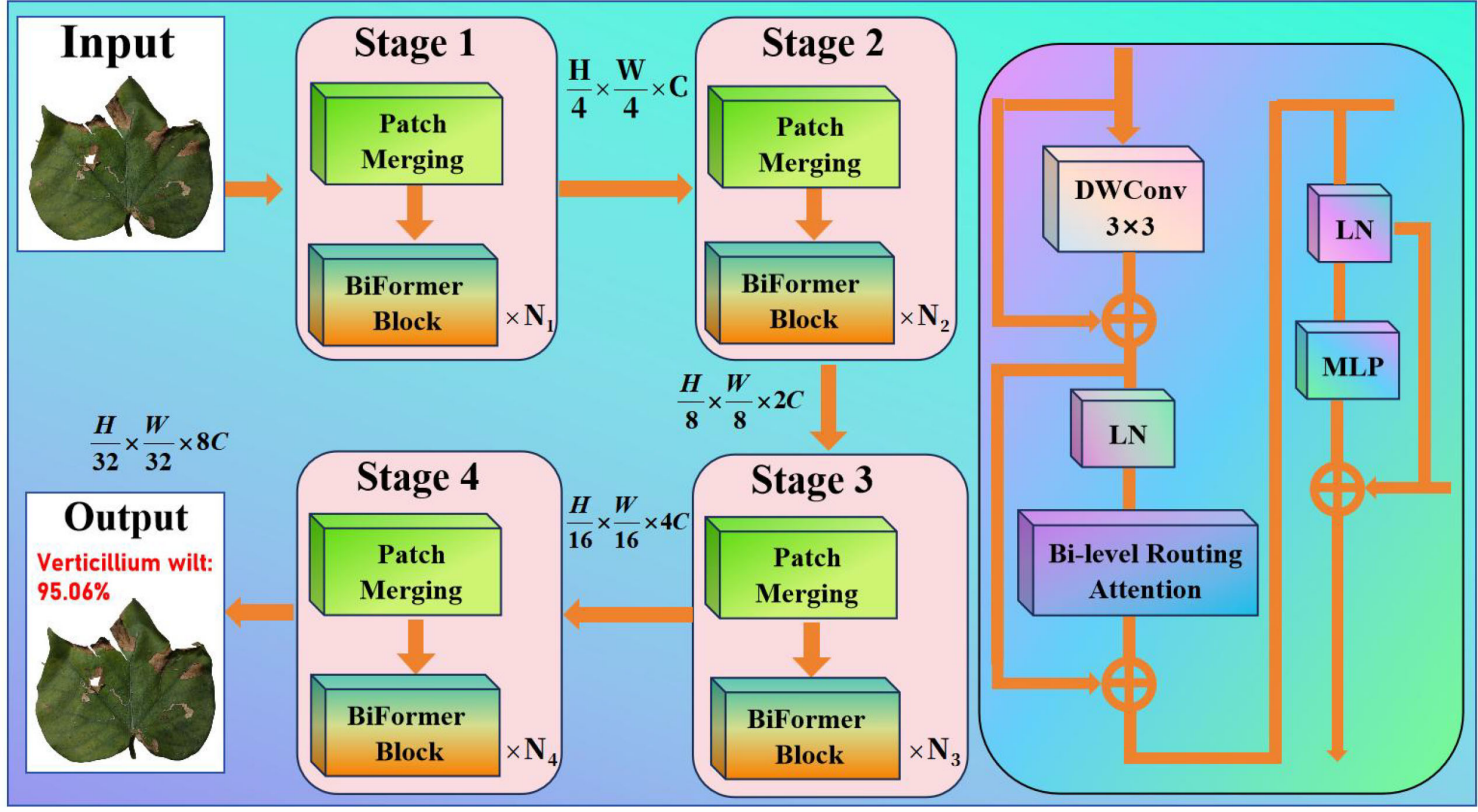
Proposed BiFormer.

Although the upgraded ViT and KAN have made progress in global feature modeling and critical local pattern extraction, they are still insensitive to cross-scale, minor morphological changes and intricate spatial linkages in cotton images. Cotton disease images may contain morphological variances and background interference between people, in addition to rich local textures and fine-grained structures. Based on local feature augmentation, ViT+KAN continues to rely primarily on interlayer attention stacking for information integration, with limited ability to record complex spatial linkages, resulting in insufficient detection of several essential characteristics. The entire architecture of BiFormer is depicted in **Figure 3**.

As a result, we introduce the BiFormer module, which is built on an upgraded ViT+KAN. The BiFormer module provides fine optimization for the feature interaction stage. The feature map processed by ViT+KAN contains global contextual information and local key patterns while retaining enough spatial resolution and multi-scale structural information. The BiFormer employs a sparse dynamic attention mechanism that dynamically identifies and prioritizes key feature regions, performing computations only between these selected areas. This approach reduces the computational complexity of traditional Transformers from quadratic to nearly linear. Consequently, when processing high-resolution cotton disease images, the model maintains high classification accuracy while achieving substantial gains in computational efficiency and reductions in memory usage. At this point, the BiFormer module can improve bidirectional information exchange across spatial regions, effectively integrate local details and global structural features of cotton, suppress redundant information interference, and allow the model to better focus on key feature regions for cotton species differentiation, thereby improving classification performance and recognition accuracy.

Assuming a two-dimensional input feature map $\text{X}\in {\mathbb{R}}^{\text{H}\times \text{W}\times \text{C}}$ is given, we divide it into S×S non-overlapping regions, each containing $\frac{HW}{S^{2}}$ feature vectors. This step is achieved by reshaping X into $X^{r}\in {\mathbb{R}}^{S^{2}\times \frac{HW}{S^{2}}\times C}$. Obtain the query Q, key K, and value V through linear projection. The process can be defined Equation 17:

(17) $$Q=X^{r}W^{q},K=X^{r}W^{k},V=X^{r}W^{v}$$

where $W^{q}$, $W^{k}$, and $W^{v}\in {\mathbb{R}}^{C\times C}$ are the projection weights of Q, K, and V, respectively.

Build a directed graph to determine the other regions that each region should focus on. First, calculate the region level queries $Q^{r}$ and keys $K^{r}$ by taking the average of the queries and keys for each region. Next, calculate the adjacency matrix $A^{r}$ and obtain it by matrix multiplication of $Q^{r}$ and $K^{r}$. It can be defined by Equation 18:

(18) $$A^{r}=Q^{r}{\left(K^{r}\right)}^{T}$$

The elements in the adjacency matrix $A^{r}$ represent the semantic correlation between two regions. Then, by preserving the first k connections of each region to trim the graph, the index matrix $I_{r}\in N^{S^{2}\times k}$ is obtained using the row-by-row topk operation. It can be defined by Equation 19:

(19) $${\text{I}}^{\text{r}}={\text{ topkIndex(A}}^{\text{r}})$$

The *i*-th row of the index matrix ${\text{I}}^{\text{r}}$contains the indices of the k regions most relevant to the i-th region. We use the region-to-region routing index matrix ${\text{I}}^{\text{r}}$ to achieve fine-grained token-to-token attention. For each region i, we collect keys and values related to that region. It can be expressed by Equation 20:

(20) $$K^{g}=gather(K,I^{r}),V^{g}=gather(V,I^{r})$$

Finally, we apply an attention mechanism to the collected key value pairs. It can be determined Equation 21:

(21) $$O=Attention(Q,K^{g},V^{g})+LCE(V)$$

where LCE (V) is a local context enhancement term implemented through deep convolution.

**Figure 4:**
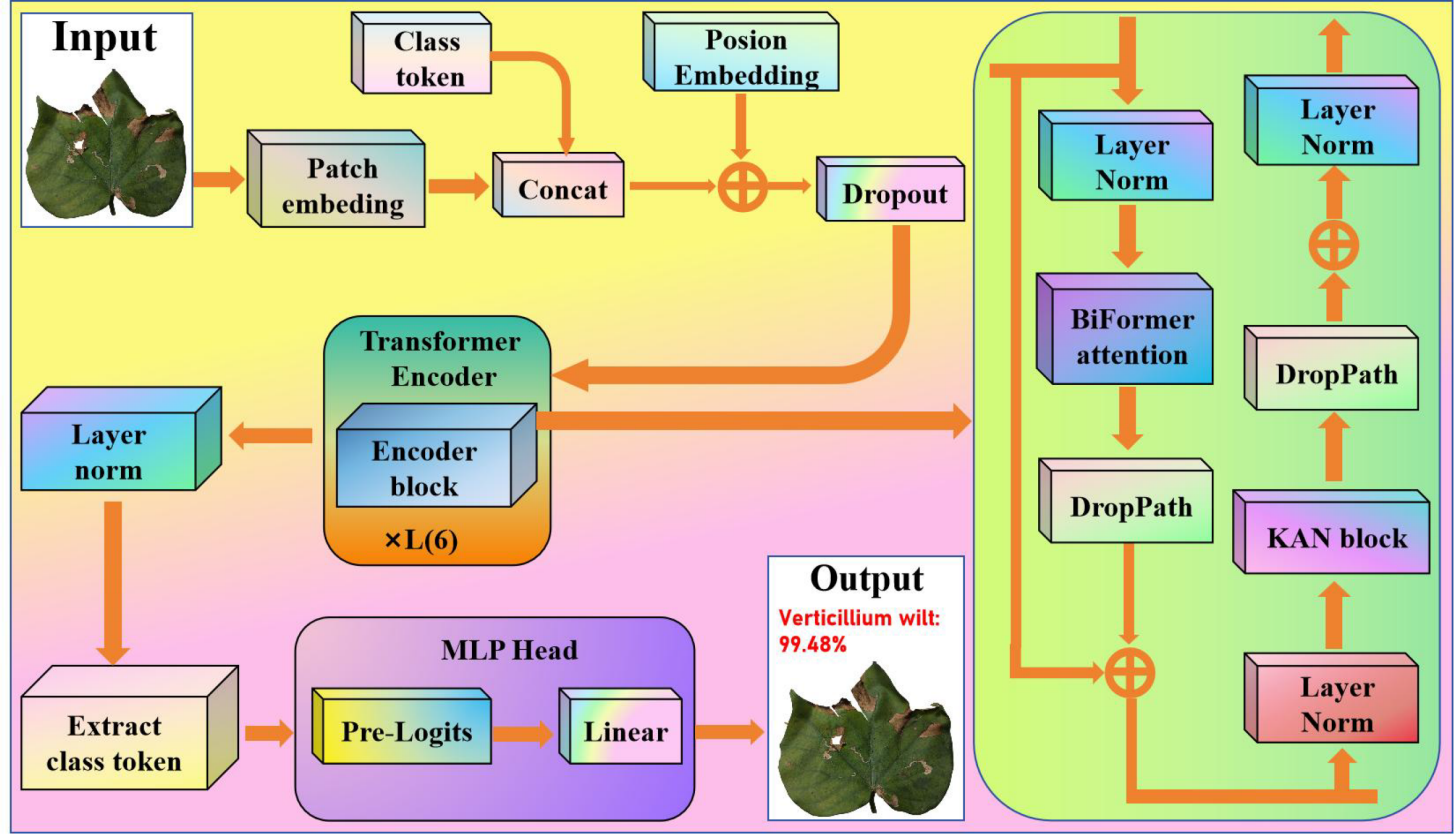
Proposed overall cotton spectral classification framework.

Based on the above analysis, the proposed overall cotton spectral classification framework is sketched in **Figure 4**. First, we simplify the Vision Transformer to improve the model’s inference speed. Second, in each Transformer layer, we use BiFormer attention to calculate dynamic sparse attention, preserving local attention to cotton feature regions in shallow levels and focusing on cotton’s global links in deep layers. Finally, we employed KAN to improve the nonlinear representation by dynamically adjusting the feature transformation intensity at various depth layers and enhancing sensitivity to minor changes in cotton color and texture. The integration of the Kolmogorov-Arnold network and BiFormer attention mechanism substantially improves the model’s robustness against variations in lighting and background interference. By employing learnable spline functions, the KAN accurately captures complex texture patterns, thereby effectively mitigating disturbances caused by non-uniform illumination. Meanwhile, The BiFormer’s sparse attention mechanism dynamically focuses on key disease-affected regions while filtering out irrelevant background noise. Through their synergistic interaction, the model achieves stable and reliable recognition performance even in complex field environments. This enabled the model to maintain efficient feature extraction while speeding up the convergence process, hence enhancing generalization ability and resilience.

## 4 Experimental results and analysis

### 4.1 Experimental setup

We created a dedicated experimental environment, and **Table 1** lists precise configuration details such as the deep learning framework version, development tools, and testing programming language. The experimental model was initialized from a pre-trained Vision Transformer. First, the model was trained as a classifier until it achieved better performance. The trained parameters were subsequently saved as a weight file. Finally, the model was evaluated on a separate, unseen test dataset by loading the pre-trained weights.

**Table 1:** Experimental setup.

Operating system	Windows 11(64-bit)
Graphics card	Intel Iris Xe Graphics
Central processing unit	Intel Core i5-13500H
Memory	32GB
Deep learning framework	Pytorch 2.4.1
Editor	PyCharm 2024.2.3
Programminglanguage	Python 3.9
Operating system	Windows 11(64-bit)

To identify the optimal experimental settings for key parameters (e.g., learning rate, epoch, batch size, and optimizer type), we evaluated the proposed model under various configurations. The results are presented in **Table 2**.

**Table 2:** Comparison of test results with different parameter configurations.

Learning rate	Epoch	Batch size	Optimizer	Test loss	Test accuracy
0.001	50	64	Adam	0.9687	96.33%
0.001	100	32	Adam	0.0685	97.36%
0.001	150	16	Adam	0.0744	97.79%
0.0001	50	16	Adam	0.0865	97.78%
0.0001	150	32	Adam	0.6556	97.82%
0.0001	**100**	**64**	**Adam**	**0.0265**	**98.05%**
0.0002	50	64	Adam	0.1243	98.01%
0.0002	100	32	Adam	0.0434	97.73%
0.0002	150	16	Adam	0.0792	97.56%
0.001	50	64	Adagrad	0.9699	95.34%
0.001	100	32	Adagrad	0.0768	96.27%
0.001	150	16	Adagrad	0.0868	96.52%
0.0001	50	16	Adagrad	0.0978	96.98%
0.0001	150	32	Adagrad	0.7686	96.92%
0.0001	100	64	Adagrad	0.0357	97.85%
0.0002	50	64	Adagrad	0.2126	97.02%
0.0002	100	32	Adagrad	0.0567	95.56%
0.0002	150	16	Adagrad	0.0876	96.88%

The bold values are used to highlight that they are optimal compared to other numerical combinations. They are also the final parameter settings used in the model we proposed.

As shown in **Table 3**, the testing accuracy and loss vary considerably across different parameter configurations. The specific combination of a learning rate of 0.0001, 100 epochs, a batch size of 64, and the Adam optimizer demonstrates superior performance compared to other settings. Consequently, this combination was selected for the model.

**Table 3:** The specific distribution of original and augmented samples.

Categories	Brown spot	Verticillium wilt	Healthy	Fusarium wilt
Original	609	516	555	672
Augmented	943	773	832	1008
Total number	1552	1289	1387	1680

To mitigate overfitting, we implemented several regularization techniques. A dropout layer (rate=0.5) was incorporated in fully connected layers to randomly disable neurons during training. L2 regularization (weight decay) was added to constrain model complexity, while batch normalization was applied to stabilize the learning process. We also employed early stopping based on validation performance to prevent overtraining. For model evaluation, 5-fold cross-validation was adopted to obtain robust performance estimates.

### 4.2 Data partitioning

**Figure 5:**
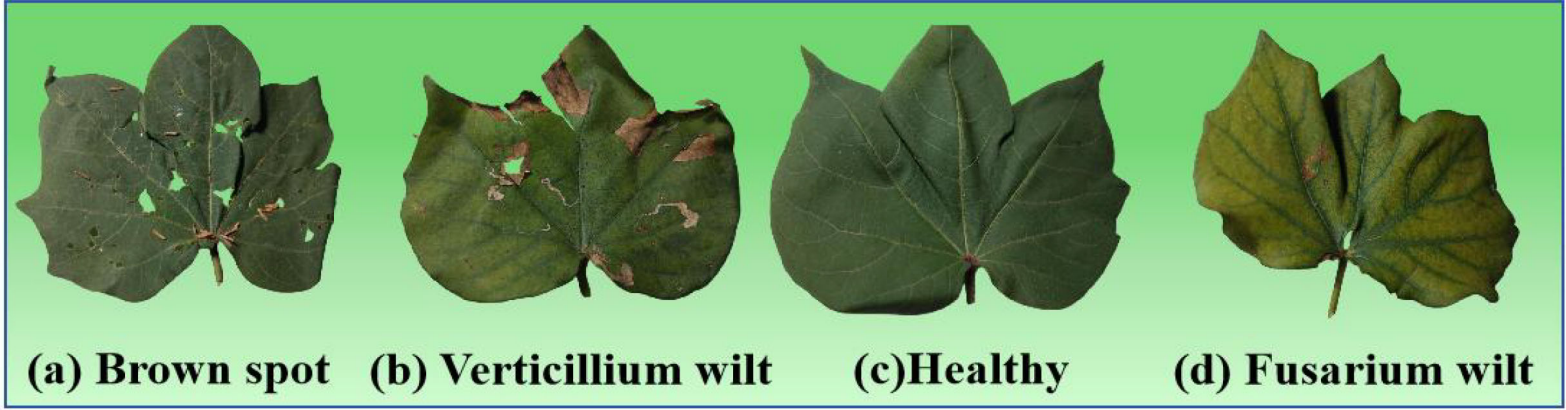
Four samples. **(a)** usually presents as spindle shaped lesions. **(b)** has more discoloration at the edges. **(c)** keeps uniform and consistent color. **(d)** is severe on the entire leaf.

The experimental data were obtained from the cotton planting bases of Henan Province, China. Based on a comprehensive review of the literature, field investigations, and consultations with cotton disease experts, we have identified four categories for this study: brown spot, verticillium wilt, fusarium wilt, and healthy cotton plants. These three selected diseases are highly prevalent and damaging in the major cotton-producing regions of Henan. There were 5,908 samples in total, mostly healthy and three disease categories: brown spot, verticillium wilt, fusarium wilt, and wheel. Four samples are shown in **Figure 5**.

To address the limited sample size of cotton disease images, we employed the Pix2Pix generative adversarial network along with conventional data augmentation techniques including rotation, scaling, and flipping to enlarge the dataset. Pix2Pix generates highly realistic and diverse synthetic samples derived from the original images, with variations in lesion morphology, lighting conditions, and background environment. This approach helps expand the high-quality training data, thereby improving the model’s generalization capability and robustness. The distribution of original and augmented samples across four types of cotton leaf diseases is presented in **Table 3**.

We randomly divided the 5908 samples into the training, validation, and testing sets with a ratio of 3:1:1. The quantity distributions are listed in **Table 4**.

**Table 4:** The division of the experimental data.

Categories	Training set	Validation set	Testing set	Total number
Brown spot	932	310	310	1552
Verticillium wilt	775	257	257	1289
Healthy	833	277	277	1387
Fusarium wilt	1008	336	336	1680
Total number	3548	1180	1180	5908

### 4.3 Experimental results

**Figure 6:**
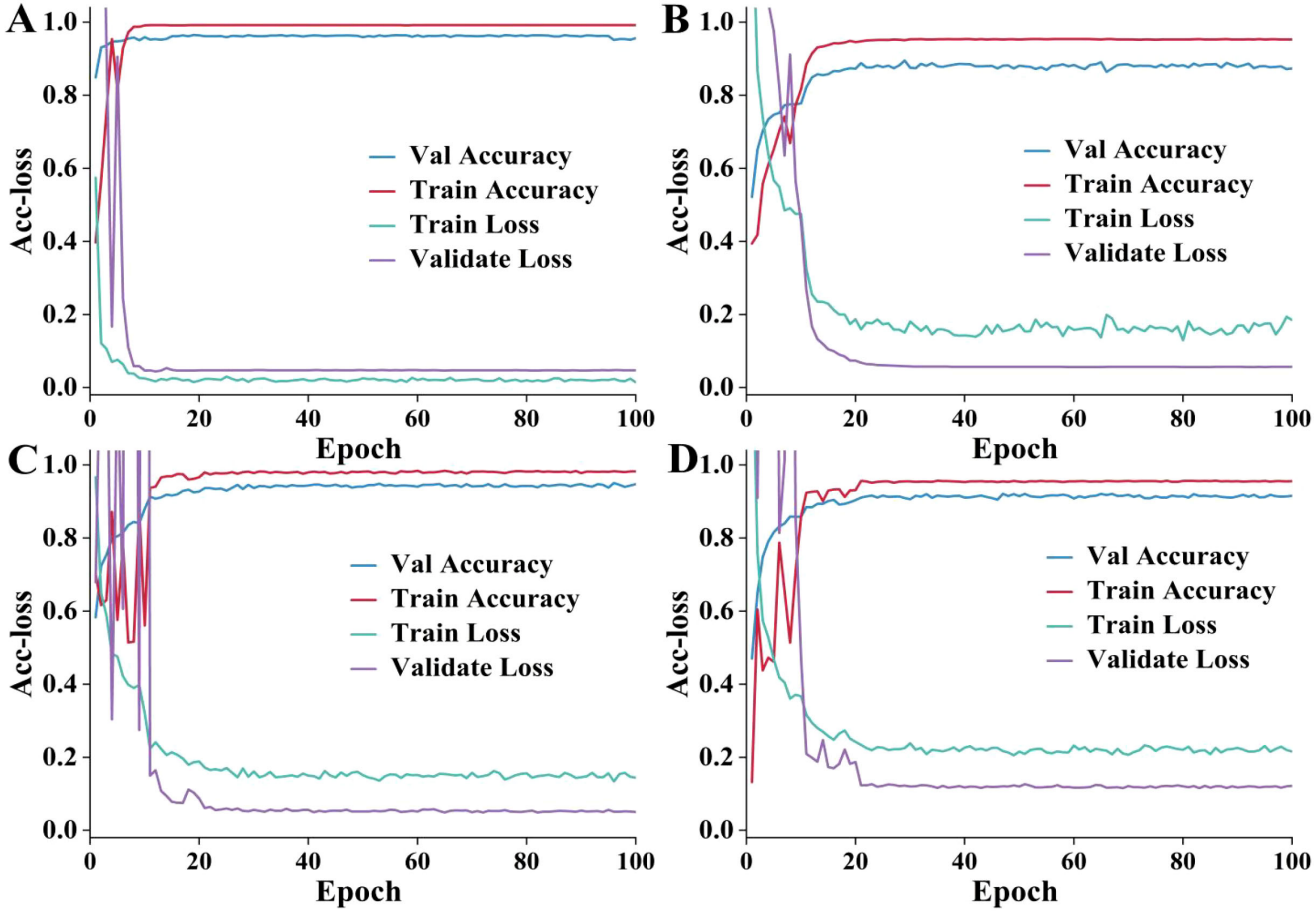
The accuracy and loss (Acc-loss) curves. **(A–D)** are the improved VIT+KAN+BiFormer, improved VIT+KAN, improved VIT+BiFormer, classical VIT, respectively. **(A)** has a better training effect. **(B)** has fluctuations in validation accuracy and training loss. **(C)** fluctuates significantly in the first 20 epochs. **(D)** is weaker than the other three methods.

To compare the performance of different approaches in the training and validation process, a trend analysis of training accuracy, training loss, validation accuracy, and validation loss was performed. The results are delineated in **Figure 6**. The improved ViT+KAN+BiFormer exhibits higher accuracy and lower loss values during both training and validation phases. It has a fast convergence speed and excellent stability. Compared with other ablation combination models, the proposed model has the best performance on the validation set. It indicates that the fusion of the KAN and BiFormer modules can effectively enhance feature extraction and discrimination capabilities. The improved ViT models incorporating the BiFormer and KAN modules alone achieved stable training accuracies of approximately 0.975 and 0.905, respectively. The superior accuracy attained with the BiFormer module suggests that it contributes more significantly to the model’s performance than the KAN module. The improvement of adding the KAN alone is lower than that of the BiFormer. The result indicates that BiFormer has a greater performance contribution. Overall, the proposed model achieves higher feature extraction capability and faster training results while ensuring a lower number of parameters, providing an efficient and reliable solution for cotton leaf disease identification.

In order to clearly observe the classification performance of the proposed method for each type of algae, we compared it using five indicators. The results are sketched in **Table 5**.

**Table 5:** Evaluation index results for four types of cotton.

Category	Precision	Specificity	F1-score	Recall	Accuracy
Brown spot	0.9921	0.9891	0.9924	0.9930	0.9821
Verticillium wilt	0.9902	0.9902	0.9902	0.9906	0.9814
Healthy	0.9892	0.9881	0.9894	0.9901	0.9762
Fusarium wilt	0.9931	0.9891	0.9936	0.9944	0.9806

From **Table 5**, it can be seen that the proposed model performs well in identifying different categories of cotton leaf diseases. For brown spot, its F1 score reaches 0.9924. This indicates that the proposed model performs well in extracting edge and color texture features of lesions. The various indicators of Verticillium wilt classification are all around 0.99. This indicates that the proposed model can effectively identify leaf wilting and chlorosis characteristics. In the Healthy category, although each indicator is slightly lower than the disease category, precision and recall are both close to 0.99. This demonstrates the model’s excellent discriminative ability. The classification performance of Fusarium wilt is the best. It indicates that the proposed model can fully capture the characteristics of leaf tissue necrosis and yellowing. Overall, the proposed model has high classification performance in identifying various cotton leaf diseases and can achieve stable and reliable disease classification.

We conducted ablation experiments and other outstanding models on parameters (Params), floating-point operations (FLOPs), and frames per second (FPS) in **Table 6**.

**Table 6:** Comparison of different models on Params, FLOPs and FPS.

Algorithms	Params(M)	FLOPs(M)	FPS
CLIP	102(↑44.4)	14800(↑3600)	5.8(↓24.2)
PaLI	17000(↑16942.4)	25000(↑13800)	20(↓10.0)
CoAtNet-7	24400(↑24342.4)	12500(↑1300)	50(↑12.0)
Classical ViT	57.6	11200	30
Improved ViT	48.0(↓9.60)	8800(↓2400)	38 (↑8.0)
Improved ViT+KAN	49.0(↓8.60)	9000(↓2200)	39 (↑9.0)
Improved ViT+BiFormer	47.3(↓10.3)	8333(↓2867)	42 (↑12)
Improved ViT+KAN+BiFormer	48.6(↓9.00)	8533(↓3067)	44 (↑14)

The arrow pointing upwards “↑” indicates an increase, while the arrow pointing upwards “↓” indicates a decrease.

As presented in **Table 6**, the improved ViT+KAN+BiFormer model surpasses all ablation combinations and the three outstanding deep learning models across all three evaluation metrics. In terms of parameters, the proposed model is only 1.3M larger than the ViT+BiFormer variant. Notably, it achieves a 2.4-fold reduction in FLOPs compared to CoAtNet-7. While the standalone KAN module exhibits higher parameter and computational costs than the standalone BiFormer, their integration yields a highly efficient architecture. Although the proposed model’s FPS is 6 lower than the top-performing CoAtNet-7, its parameter count is substantially smaller. Overall, our model offers an optimal balance of high speed and low computational complexity.

**Figure 7:**
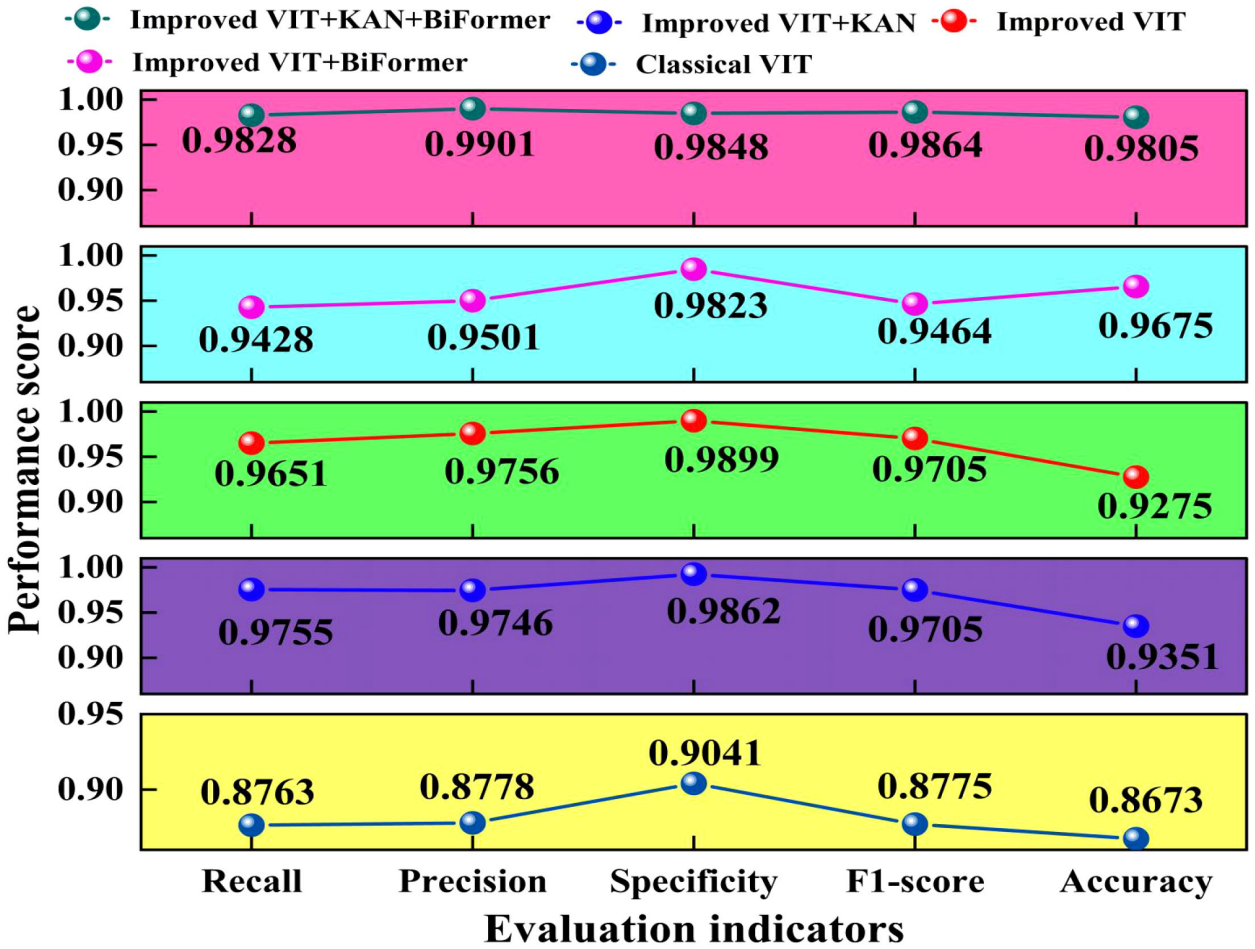
Comparison of different performance evaluation indicators in ablation studies.

To verify the effectiveness of the improved ViT core modules, we used five evaluation indicators to perform ablation experiments. The results are depicted in **Figure 7**.

As illustrated in **Figure 7**. The improved ViT+KAN+BiFormer outperforms the other four ablation combination models in all five evaluation metrics. Compared with the improved ViT+BiFormer, the proposed model improves recall, precision, and F1-score by about 4.0%. Compared to the traditional ViT, the improved ViT incorporating the BiFormer and KAN modules achieved accuracy gains of 10.2% and 6.78%, respectively. The more substantial improvement observed with the BiFormer module suggests it contributes more significantly to performance enhancement than the KAN module. This indicates that the synergistic effect of the KAN and BiFormer significantly enhances the feature expression ability and generalization performance of the model. Overall, the proposed model has high recognition accuracy and stronger robustness in various indicators, providing an efficient and reliable model solution for identifying cotton leaf diseases.

**Figure 8:**
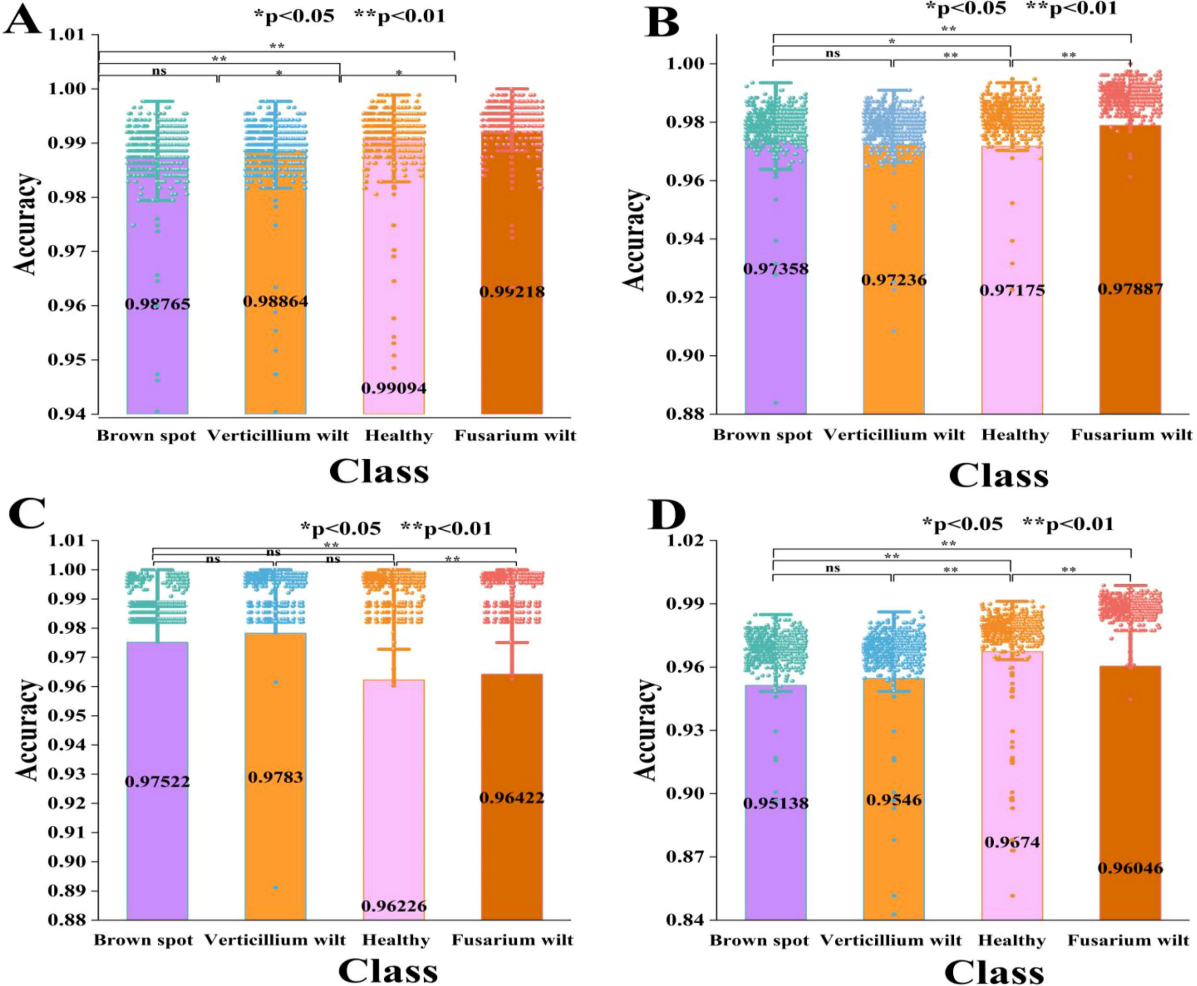
A bar scatter plot with significant differences in the ablation experiment. **(A)** is Improved VIT+KAN+BiFormer, **(B)** is Improved VIT+KAN, and **(C)** is Improved VIT+BiFormer, and **(D)** is Classical VIT.

A bar-scatter plot with significant differences serves as an effective visual tool for evaluating module efficacy and quantifying contributions. It displays the average performance of different models via bar height while incorporating scatter points and error bars (95% confidence interval) to reveal dispersion and statistical variability of individual predictions, thus reducing random fluctuations. Significance markers (*p<0.05, **p<0.01) were applied to verify that performance improvements are statistically substantial. As shown in **Figure 8**, the average accuracy, scatter distribution, error ranges, and significance levels across four cotton varieties are systematically compared.

As illustrated in **Figure 8**, the improved ViT+KAN+BiFormer achieved the highest accuracy on four types of cotton leaf disease samples. Its overall performance is superior to the other three ablation combination models. It is about 1.5%, 1.7%, and 3.5% higher than the improved ViT+KAN, Improved ViT+BiFormer, and Classical ViT, respectively. From the comparison of various categories, the proposed model shows the most significant improvement in the recognition of healthy leaves and Fusarium wilt. This indicates that the fusion of KAN and BiFormer can effectively enhance the model’s ability to perceive subtle disease features and differences from normal tissues. Overall, the improved ViT+KAN+BiFormer model has stronger feature expression ability and better classification stability, providing a high-precision and robust technical solution for cotton leaf disease recognition.

**Figure 9:**
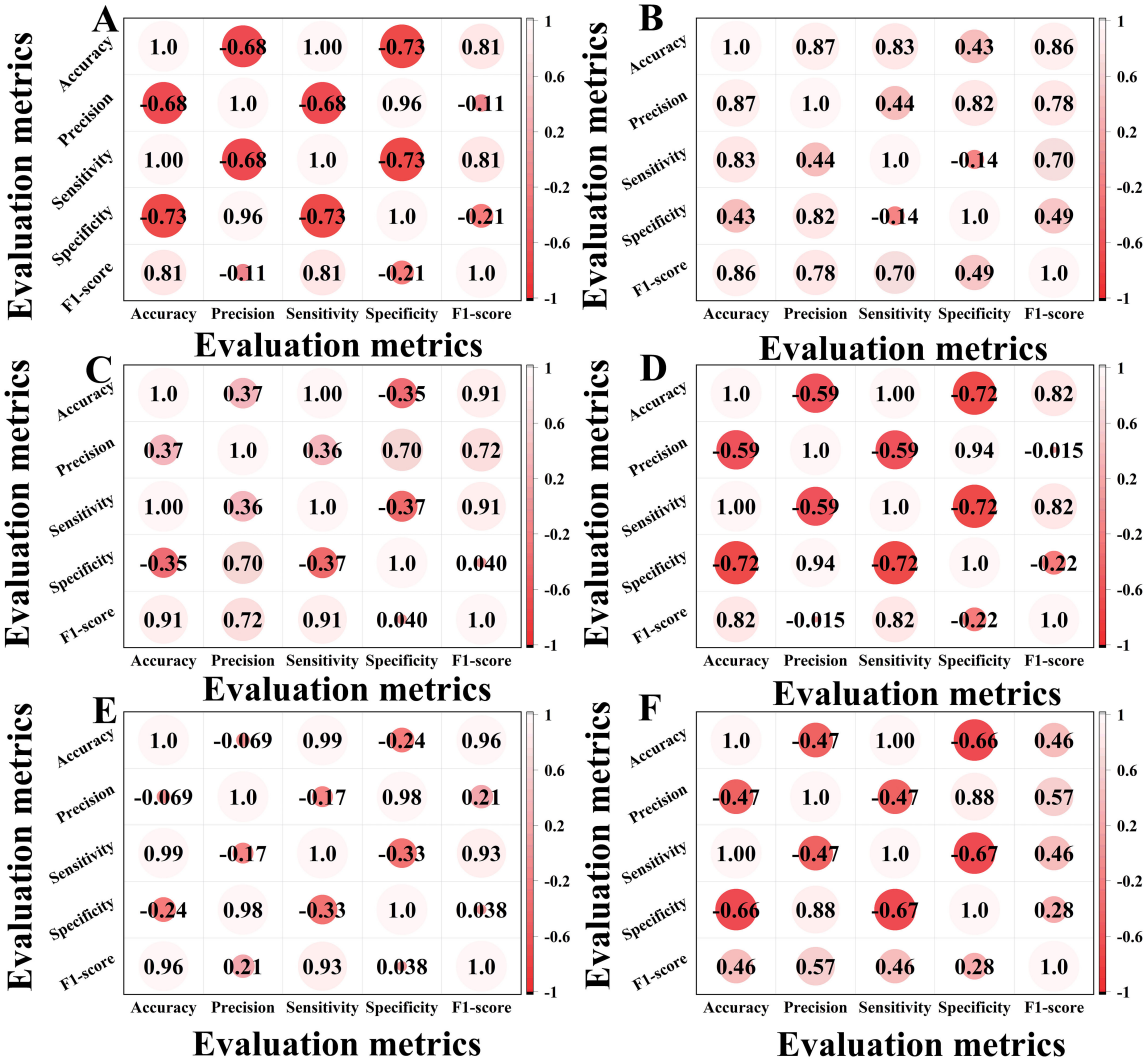
Correlation heatmaps of ablation experiments. **(A)** is a correlation heatmap of the classical ViT. **(B)** is a correlation heatmap of the classical VIT+KAN+BiFormer. **(C)** is a correlation heatmap of the improved VIT. **(D)** is a correlation heatmap of the improved VIT+KAN. **(E)** is a correlation heatmap of the improved VIT+BiFormer. **(F)** is a correlation heatmap of the improved VIT+KAN+BiFormer.

The correlation heatmap in **Figure 9** effectively visualizes relationships between evaluation metrics, revealing synergies and conflicts across assessment dimensions. Through color gradients and numerical values, it quantitatively reveals the model’s intrinsic patterns in feature learning and classification, providing visual evidence for evaluating architectural improvements in our ablation studies.

In **Figure 9**, A is the classical ViT, B is the classical VIT+KAN+BiFormer, C is the improved VIT, D is the improved VIT+KAN, E is the improved VIT+BiFormer, and F is the improved VIT+KAN+BiFormer. Some important observations can be made from the content of **Figure 9**. The improved ViT+KAN+BiFormer exhibits the highest positive correlation on all five evaluation metrics. Its overall feature relationship is the most closely related, indicating that the performance indicators within the model have the strongest synergy and the most stable performance. Compared with other models, the improved ViT+KAN+BiFormer has significantly better overall performance than Improved ViT, Improved ViT+KAN, and Improved ViT+BiFormer. This indicates that the fusion of KAN and BiFormer modules can effectively enhance feature extraction and classification consistency. Overall, the improved ViT+KAN+BiFormer, has the strongest correlation and the best overall performance, combining high accuracy and efficiency.

**Figure 10:**
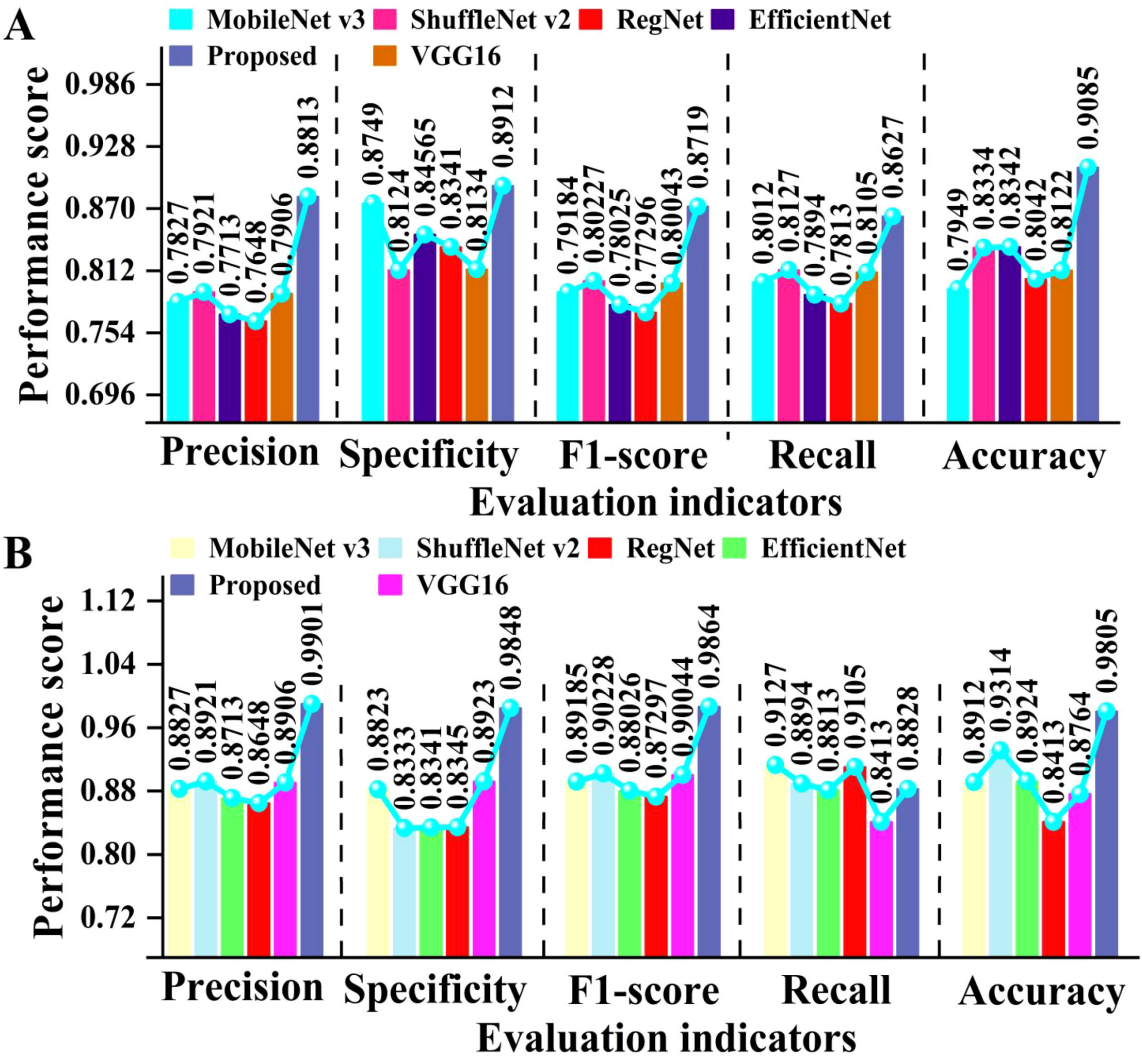
Comparison of evaluation metrics for cotton classification using several forms of deep learning. **(A)** shows the results of several models on open-source datasets, while **(B)** shows the results of several models on our collected datasets.

To verify the generalization ability of the proposed model, we tested different deep learning models on both open-source datasets and our own dataset. The test results are presented in **Figure 10**.

As can be seen in **Figure 10**, the proposed model achieved high performance on both open-source and self-built datasets. On open-source datasets, the proposed model achieved an average improvement of approximately 8.3% and 10% compared to Transformer and VGG16, respectively. On the self-built dataset, the proposed model achieved an average improvement of about 8.9% compared to EfficientNet. This fully demonstrates that the proposed model has stronger feature extraction and generalization capabilities. Overall, the proposed model has high classification performance and generalization ability on different datasets, providing an efficient and reliable solution for identifying cotton leaf diseases.

**Figure 11:**
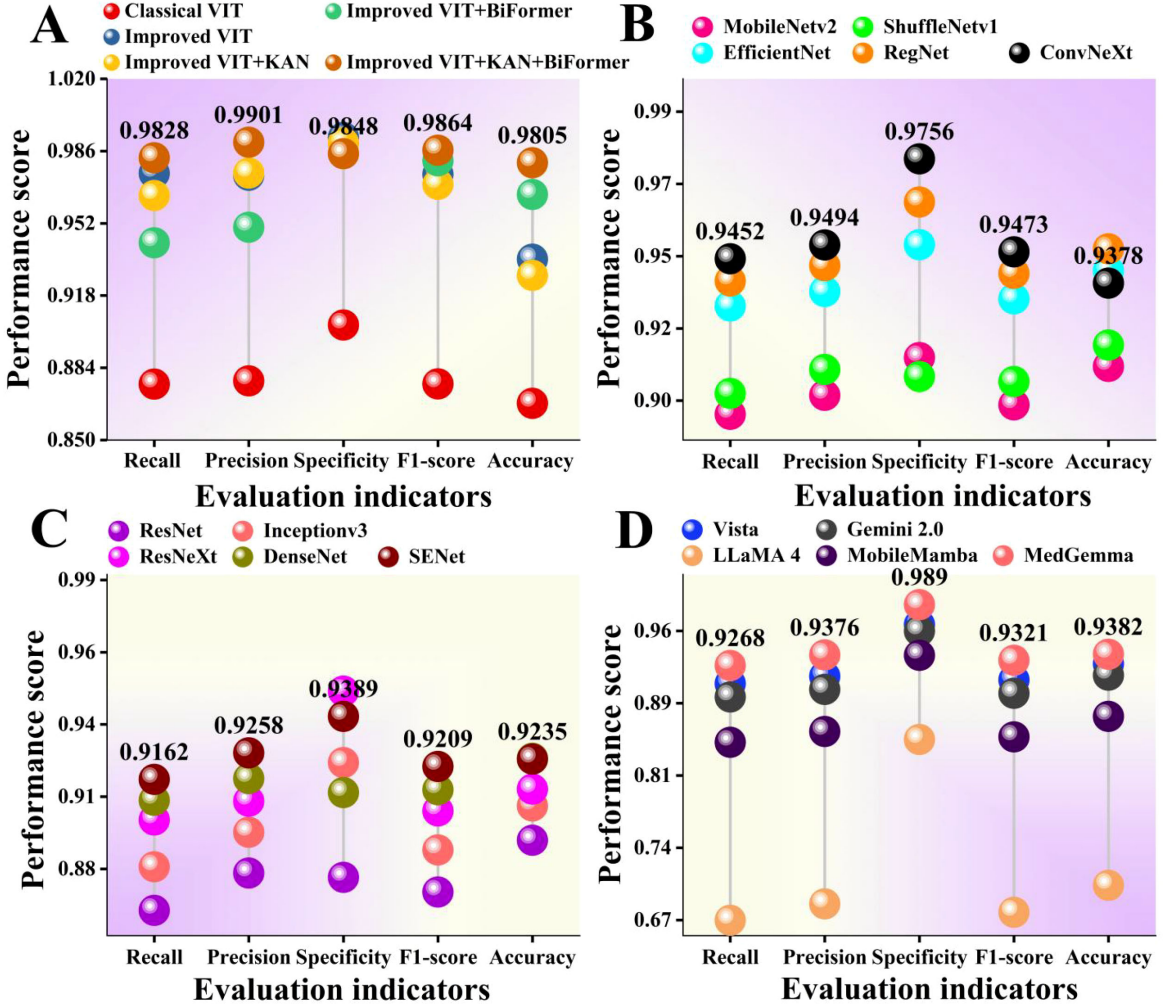
Comparison of different models. **(A)** is the ablation experimental results of our proposed model. **(B-D)** show the performance of different models on the wheat leaf disease data set we collected.

In order to comprehensively evaluate the detection performance of different models, we compared and analyzed them using lollipop plots. The lollipop chart visually presents the numerical differences between different categories through the position of dots and the length of line segments. It can clearly demonstrate the differences between multiple models on different evaluation metrics. The results are manifested in **Figure 11**.

As shown in **Figure 11** that there is a significant performance difference among the four types of deep learning models. The comprehensive performance of the improved ViT+KAN+BiFormer is the best. In terms of accuracy, the proposed model is 0.1037 higher than traditional ViT. On Recall, the proposed model is 0.0378 higher than the lightweight network ConvNeXt. On the F1 score, the proposed model is 0.0655 higher than ResNeXt. On Precision, the proposed model is 0.0525 higher than MedGemma. After integrating the KAN and BiFormer mechanisms, ViT improved the main indicators by an average of 3% -6% compared to traditional models. Overall, the proposed model has high robustness.

**Figure 12:**
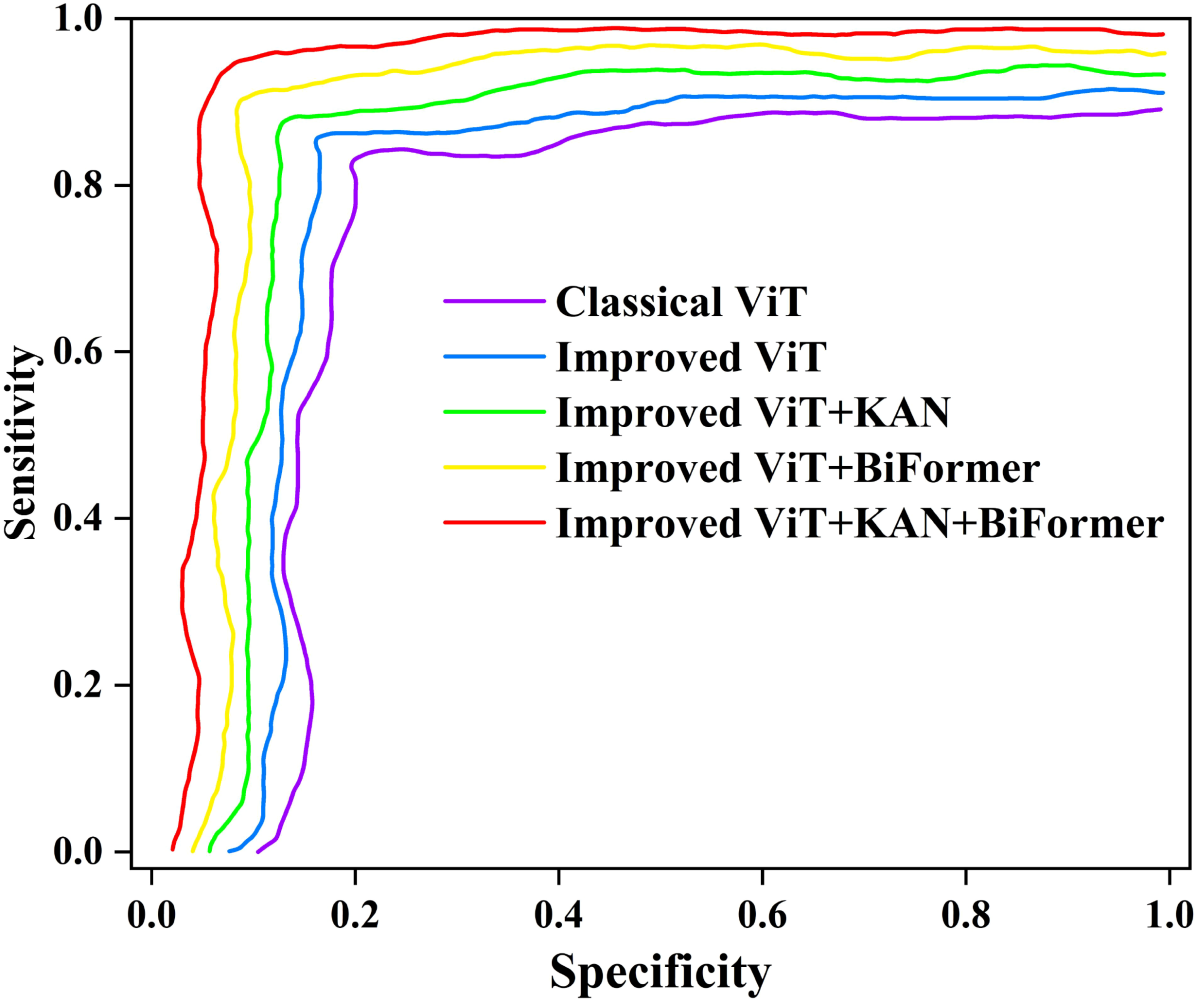
The ROC curves of different ablation experiments.

To comprehensively evaluate the performance of the classical Vision Transformer (ViT) and its improved variants in cotton leaf disease identification, particularly regarding potential biases from class imbalance, we plotted the receiver operating characteristic (ROC) curves for each model, as shown in **Figure 12**. The ROC curve depicts the relationship between sensitivity (Y-axis) and 1-specificity (X-axis). The models compared include the classic ViT, improved ViT, improved ViT+KAN, improved ViT+BiFormer, and improved ViT+KAN+BiFormer.

From **Figure 12**, we can see that the ROC curves of all five models exhibit a steep ascent in the high-sensitivity region, yet demonstrate pronounced divergence in the low-specificity range. This indicates strong overall classification capability, with variations in handling minority classes and boundary samples. The standard ViT, with its lower curve, shows limited performance on complex or imbalanced data. The improved ViT variant achieves gains in low specificity, suggesting enhanced feature extraction from structural optimizations. Performance is further improved with the incorporation of KAN, while the BiFormer-integrated model ensures greater stability in high-sensitivity areas. The complete model, combining both KAN and BiFormer, produces a curve closest to the upper-left corner, maintaining high sensitivity across the entire specificity spectrum and achieving optimal classification performance. In summary, the integration of KAN and BiFormer significantly boosts minority-class recognition and overall robustness, effectively mitigating performance degradation due to class imbalance.

**Figure 13:**
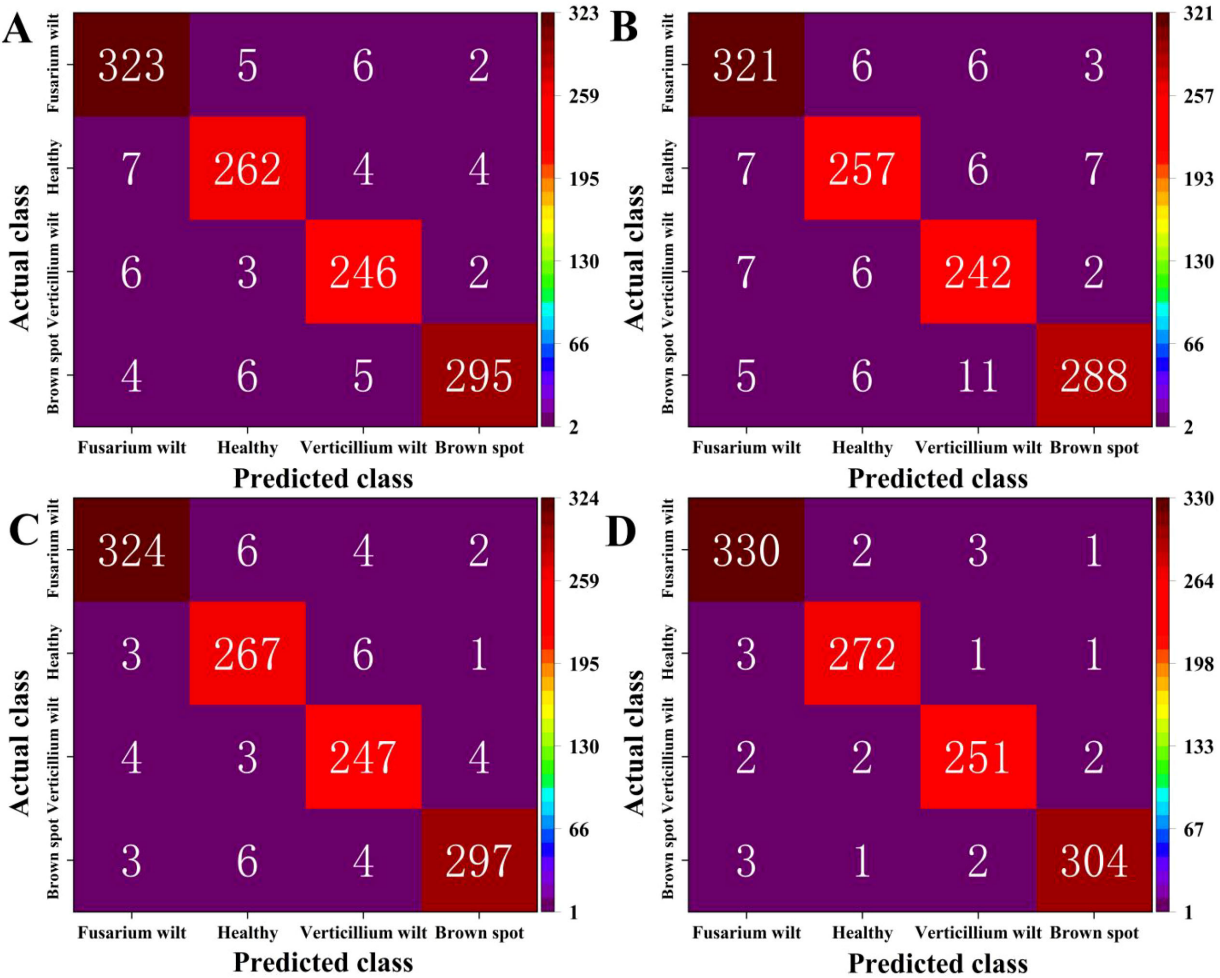
Identification results of four algae using the confusion matrix of the proposed model, where **(A)** is CoAtNet-7, **(B)** is CLIP, **(C)** is PaLI, and **(D)** is proposed.

A confusion matrix is a useful tool for determining the accuracy of supervised learning categorization. Each row of the matrix refers to the sample’s real category, and its row sum indicates the total number of that category in the test set. By using the confusion matrix, it is possible to visually compare the true categories of the samples with the predicted results of the model, identify categories that are easily confused during the classification process, and evaluate the performance of the model on each category. The confusion matrix for identifying the spectral data of four types of algae on the test set is exhibited in **Figure 13**.

From **Figure 13**, we can calculate that the overall classification accuracy of CoAtNet-7, CLIP, PaLI, and the proposed model is 95.40%, 93.80%, 96.10%, and 98.05%, respectively. Compared with the lowest CLIP, the proposed model improves by 4.25%. Compared with the highest PaLI, the proposed model improves by 1.95%. There is not much difference between CoAtNet-7 and PaLI, indicating that they have similar performance in classifying cotton leaf diseases. Overall, the proposed model has high classification and recognition performance for cotton leaf diseases.

**Figure 14:**
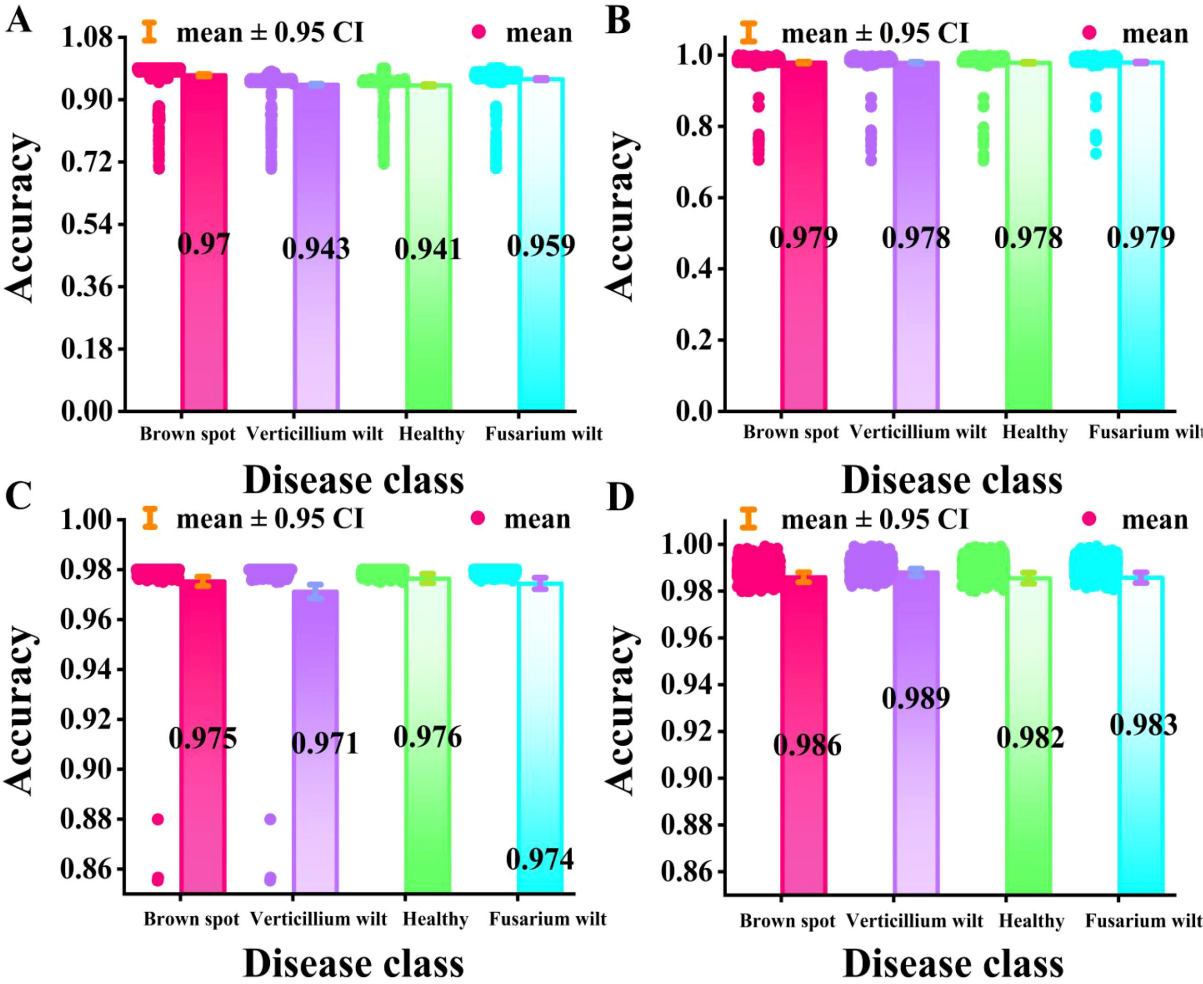
The mean error test results of the model’s recognition accuracy. **(A)** is RegNet, and the average confidence level is above 94.1%. **(B)** is ConvNeXt, and the average confidence level is above 97.8%. **(C)** is SENet, and the average confidence of the four methods is above 97.1%. **(D)** The proposed approach in this paper, with average confidence exceeding 98.2%.

Interval plots are visualization tools that display the range of data variation and estimation accuracy through boundary values such as start and end points and confidence intervals. **Figure 14** compares the probability confidence level of this method with other combination methods in the test results and presents the results of significance tests for the mean error of the 95% confidence interval. The bar chart depicts the average accuracy of each method in identifying different cotton categories, with error bars representing the 95% confidence interval around the mean for different models.

From **Figure 14**, it can be seen that the average confidence score of the proposed model is better than the other three models. Compared with the lowest RegNet, the proposed model improves by 4.1%. Compared with the highest ConvNeXt, the proposed model improves by 0.4%. The average confidence scores of ConvNeXt and SENet are not significantly different, indicating that they have similar recognition performance for cotton leaf diseases. Overall, the proposed model has significant advantages in cross-category discrimination and generalization ability and can accurately identify diverse categories of cotton leaf diseases, providing a more reliable technical solution for cotton leaf disease identification.

**Figure 15:**
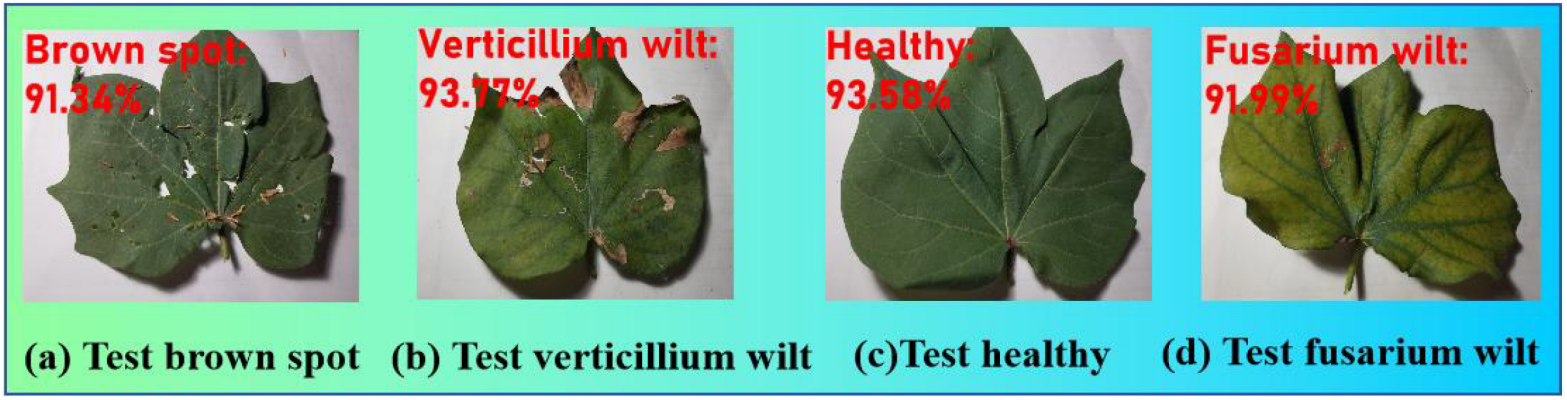
The testing results with the proposed model. **(a)** was classified correctly with 91.34% confidence. **(b)** was classified correctly with 93.77% confidence. **(c)** was classified correctly with 93.58% confidence. **(d)** was classified correctly with 91.99% confidence.

We use the proposed deep learning model to randomly select one from the test set for testing. **Figure 15** presents the predicted results. The confidence level of each recognition probability is greater than 90%.

To evaluate the generalization capability of the proposed ViTKAB model across different crops and plant diseases, we conducted experiments on four publicly available datasets (Zhao et al., 2025): the Cucumber Plant Diseases Dataset, Rice Diseases Image Dataset, Plant Pathology Apple Dataset, and Corn Leaf Diseases Dataset. These datasets were compared with our original cotton leaf disease data. The results are summarized in **Table 7**.

**Table 7:** Comparisons of the five different datasets.

Datasets’ names	Precision	Specificity	F1-score	Recall	Accuracy
Cucumber Plant Diseases Dataset	0.9821	0.9791	0.9825	0.9831	0.9721
Rice Diseases Image Dataset	0.9802	0.9802	0.9801	0.9801	0.9714
PlantPathology Apple Dataset	0.9792	0.9781	0.9798	0.9806	0.9662
Corn Leaf Diseases(NLB)	0.9831	0.9791	0.9837	0.9844	0.9706
Our datasets	0.9901	0.9848	0.9864	0.9828	0.9805

As shown in **Table 7**, the proposed model demonstrates high classification performance across all four open-source datasets. On our own dataset, it achieves a precision that is 1.09% higher than the lowest value recorded on the PlantPathology Apple Dataset, and a recall that is 0.27% higher than the lowest value from the Rice Diseases Image Dataset. These results confirm the model’s strong robustness and excellent generalization capability.

### 4.4 Deployment potential on edge devices

We present the first integration of KAN and BiFormer modules into a lightweight Vision Transformer (ViT), achieving an accuracy of 98.05% on a four-class cotton leaf disease dataset. The proposed model outperforms EfficientNet and ShuffleNet variants while maintaining a moderate parameter count of 48.6M, suitable for edge deployment. To further validate the inference speed, robustness, and edge compatibility of the model, we evaluated four lightweight models in terms of parameters, FLOPs, speed, and accuracy under a consistent hardware setup (32GB memory, RTX 4090 GPU). Detailed results are provided in **Table 8**.

**Table 8:** Comparison of the proposed method with other methods.

Models’ names	Params (M)	FLOPs (M)	FPS	Accuracy
MobileNet-V3	53	217	133	94.63%
ShuffleNetv2	7.9	531	55.6	93.02%
EfficientNetV2	119.36	340	40	94.50%
ViTKAB	48.6	8533	44	98.05%

Our model achieves a classification accuracy of 98.05%, significantly surpassing other compared models and satisfying core requirements for crop recognition and disease detection in agricultural applications. With a parameter size of 48.6M, it imposes minimal strain on the limited storage resources of edge devices. In terms of key deployment metrics, although the current FLOPs value of 8533M is higher than those of other lightweight models, mature edge optimization techniques such as quantization and structured pruning can effectively reduce computational overhead. The inference speed of the FPS of 44 already exceeds that of ShuffleNetv2 and EfficientNetV2. While MobileNet-V3 achieves the FPS of 133, this gap can be narrowed through further model lightweighting and inference framework adaptation, thus adequately meeting real-time requirements in agricultural edge scenarios. In summary, the ViTKAB model possesses the essential attributes for edge deployment, including high recognition accuracy and a manageable parameter scale. Future work will focus on optimizing FLOPs and inference speed to facilitate efficient deployment on agricultural edge devices.

The method introduced in this study offers distinct advantages for edge device deployment. Based on a lightweight and efficient ViT architecture, we have for the first time effectively combined KAN and BiFormer attention mechanisms. Through parameter sharing and computational optimization, the model enhances expressive power while controlling computational costs, preserving its lightweight nature. This design enables adaptation to resource-constrained environments and supports real-time image and video processing with low latency and power consumption—key requirements for edge applications.

Looking forward, we plan to advance edge deployment in two directions: first, by adopting compression techniques such as knowledge distillation and quantization to reduce parameter size and improve inference efficiency; second, by optimizing computational workflows and developing modular deployment strategies tailored to specific edge hardware. These steps will help achieve load balancing and system stability while extending the model’s applicability to diverse edge scenarios.

## 5 Discussion

We propose a cotton disease classification method based on an enhanced Visual Transformer, KAN, and BiFormer. This technique successfully tackles cotton image recognition issues such as low sample size, feature refinement, and high inter-class similarity. We obtain cotton disease pictures using high-resolution microscopic imaging equipment and convert image characteristics into embeddings suited for Transformer processing (Holicheva et al., 2025), allowing the model to better capture cotton shape, texture, and spatial information. The analysis of the proposed model’s performance on the test set revealed distinct response patterns to different disease features, such as texture roughness and color changes. The proposed model accurately identified subtle texture variations in early-stage lesions and color migration in advanced lesions, thereby directly enhancing the classification system’s discriminatory reliability in complex backgrounds.

The KAN module uses a channel attention method to adaptively weight feature responses across multiple channels, emphasizing cotton morphological information (Rivera et al., 2025). Compared to standard ViT or single-module attention techniques, KAN delivers higher-dimensional information fusion in multi-channel feature spaces, considerably improving the model’s discrimination capacity and robustness for cotton classification. In contrast to the standard knowledge distillation approach described by Xu et al. (2025), integrating the KAN channel attention mechanism specifically concentrates on essential cotton spectral channel properties. This improves the model’s ability to capture useful information despite tiny spectral changes and complicated background interference, compensating for the inadequacies of standard feature discrimination and mining techniques. As a result, it increases the model’s classification flexibility for difficult samples.

The BiFormer module achieves deep integration of channel and spatial information through cross-layer feature interaction, enabling the model to capture multiscale fine-grained features of cotton diseases. Compared to the single modified ViT model, BiFormer effectively mitigates feature redundancy and category confusion, significantly enhancing the model’s discriminative capability in complex cotton field scenarios. By integrating KAN and BiFormer, the enhanced VIT+KAN+BiFormer model not only improves single-category recognition accuracy but also achieves significant synergistic effects across multiple indicator dimensions. Compared to the fusion attention model proposed by Yvenou et al. (2025), the BiFormer module integrated in this study significantly enhances the model’s ability to capture multi-scale features of cotton diseases. Compared to the proposed fusion attention model, the BiFormer module integrated in this study not only enhances the model’s ability to capture multi-scale features of cotton but also provides a refined feature optimization pathway for cotton recognition in complex environments through its bidirectional attention mechanism and hierarchical feature fusion design. This not only improves recognition accuracy but also provides data support and theoretical basis for adjusting the model’s scene adaptability in subsequent practical applications.

The model developed in this study demonstrates strong performance in cotton disease identification. However, potential limitations regarding overfitting and class imbalance remain. Despite employing a simplified Transformer architecture and a sparse attention mechanism, the model may still be prone to overfitting in scenarios with limited samples. This risk is particularly evident when the model learns to rely on spurious correlations such as specific background or lighting conditions in the training set rather than intrinsic pathological features. This observation aligns with the generalization challenges of deep learning models in agricultural image analysis, as noted by Liu et al. (2025), and indicates our model’s sensitivity to shifts in data distribution. Furthermore, class imbalance in the dataset may impair recognition performance for minority categories. To address these issues, future work will focus on enhancing model stability and generalization in complex real-world environments through stricter regularization, balanced data augmentation based on generative adversarial networks, and the construction of more representative validation sets.

In comparative studies, the model achieved good accuracy on four cotton categorization tasks, demonstrating the statistical reliability of multi-module collaboration. This study suggests that multi-module coordination not only improves cotton feature extraction efficiency but also promotes cross-class adaptation, resulting in a dependable model design framework for cotton and other fine-grained image classification problems.

By modeling various imaging settings and illumination interference, we discovered that the model remains very robust under extreme conditions, while performance suffers marginally in instances with extremely rare sample categories or severe occlusions. This establishes explicit guidelines for future data augmentation and architectural optimization. Future study will concentrate on increasing the diversity and complexity of the cotton disease dataset by gathering photos from various growth stages and environmental circumstances to create a more comprehensive database. Simultaneously, optimizing model design and computing complexity will improve deployment capabilities on edge devices or mobile terminals, allowing for real-time applications of the cotton categorization system in field environmental monitoring.

The model developed in this study demonstrates strong performance in identifying single leaf diseases with distinct visual symptoms, as it precisely captures the specific texture and color features associated with each condition. However, its recognition accuracy declines when dealing with early-stage diseases that lack typical symptoms, as well as cases involving co-infection by multiple diseases on a single leaf. This limitation stems from the fact that early disease characteristics closely resemble healthy features, and in multi-disease scenarios, the overlapping symptoms interfere with each other. This observation aligns with the early disease detection challenge highlighted by Gulzar and Ünal (2025), indicating that our model requires further enhancement in handling feature variability. In future work, we plan to adopt a multi-label progressive learning framework, integrate hyperspectral imaging technology, and develop a large-scale multi-label annotated dataset. These efforts will focus on improving the model’s ability to detect subclinical disease features and recognize co-occurring diseases, ultimately advancing early disease warning and accurate diagnosis of complex infections.

This study demonstrates the efficacy of structure optimization, channel refinement, and feature interaction in the problem of fine-grained cotton disease recognition with little samples. It serves as a theoretical foundation and technical reference for intelligent cotton disease monitoring and associated agricultural management. The findings show how deep learning technology may be used in a variety of applications, including smart agriculture.

## 6 Conclusions

In this article, we present a cotton disease classification approach based on an upgraded Vision Transformer (ViT), which incorporates the Kolmogorov-Arnold network (KAN) and the Feature Interaction Module (BiFormer) for feature extraction and classification of high-resolution cotton disease images. This method achieves a classification accuracy of 98.05% across four cotton disease identification tests, far surpassing classic ViT models and single-module improved models. The model achieves high synergy across all measures by employing a three-part technique of structure optimization, channel refinement, and feature interaction. Most correlation coefficients are greater than 0.8, ensuring stability and comprehensiveness in complex cotton disease identification scenarios. Compared to standard models, this approach not only reduces misclassification and missed detection rates, but it also effectively addresses the constraints given by small samples and fine-grained features, preserving structural efficiency and simplicity while avoiding overfitting.

However, the model has limitations under severe sample conditions or when gathering local edge information, and it necessitates manual adjustment of critical parameters during training. In the future, we will focus on increasing model performance using visual inspection, network design optimization, and data augmentation strategies. Furthermore, we will expand the dataset to include cotton samples from various growth stages and environmental conditions would make the model more suitable for real underwater monitoring and rapid cotton identification.

The proposed model has demonstrated strong recognition performance in laboratory settings. However, it still exhibits notable limitations in practical field deployment and scalability. For instance, the model lacks robustness to variations in lighting conditions, camera angles, and occlusions commonly encountered in complex field environments. Its generalization capability across different geographic regions and cotton varieties also requires further improvement. Furthermore, due to the limited number of training samples, the model remains susceptible to overfitting, while class imbalance may impair its sensitivity to rare diseases. To address these issues, future work will focus on developing lightweight model compression techniques to enhance computational efficiency and meet the real-time diagnostic requirements of mobile devices. We also plan to construct an open-access, multi-regional, multi-variety disease image database and employ domain adaptation methods to improve the model’s generalization. In addition, we will explore federated learning frameworks that enable continuous model optimization across distributed devices without centralizing data. These efforts aim to establish a self-evolving, distributed intelligent diagnosis system, facilitating the transition of this technology from experimental research to large-scale agricultural practice.

To summarize, the suggested model performs exceptionally well in cotton leaf disease recognition tasks due to its robustness, adaptability, and accuracy. It offers a reusable technical solution for cotton disease classification and intelligent agricultural disease monitoring, as well as a theoretical framework and practical assistance for using deep learning methods in small-sample and high-precision picture recognition.

## Data Availability

The original contributions presented in the study are included in the article/Supplementary Material. Further inquiries can be directed to the corresponding authors.
